# Opportunities for male involvement during pregnancy in Magu district, rural Tanzania

**DOI:** 10.1186/s12884-016-0853-8

**Published:** 2016-03-29

**Authors:** E. Vermeulen, A. Solnes Miltenburg, J. Barras, N. Maselle, M. van Elteren, J. van Roosmalen

**Affiliations:** Department of Medical Humanities EMGO, VU University Medical Center, Amsterdam, The Netherlands; African Woman Foundation, Mwanza, Magu District Tanzania; Project Manager Crops Marketing Bureau (CROMABU) & local Project Manager of African Woman Foundation, Magu, Mwanza, Tanzania; Department of Obstetrics, Leiden University Medical Center, Leiden, The Netherlands; Athena Institute for Research on Innovation and Communication in Health and Life Sciences, VU University, Amsterdam, The Netherlands; Department of Community Medicine, Institute of Health and Society, Medical Faculty, University of Oslo, Oslo, Norway

**Keywords:** Male involvement, Pregnancy, Maternal health, Barriers to access, Antenatal care, HIV testing

## Abstract

**Background:**

Male involvement during antenatal care is promoted to be an important intervention to increase positive maternal and new born health outcomes. Despite active promotion to stimulate male involvement during antenatal care, few men in Tanzania accompany women to their antenatal care visits. This study aims to understand perceptions, attitudes and behaviour of men regarding their role and involvement during pregnancy and antenatal care visits in a rural district in Tanzania.

**Methods:**

Data collection took place in Magu District between September 2013 and March 2014, using a mixed method approach. This included observations at six government health facilities, nine focus group discussions (with a total of 76 participants) and 26 semi-structured interviews of participants, included through convenience- and snowball sampling. Additionally, a questionnaire was distributed among 156 women attending antenatal care, regarding their partners’ involvement in their pregnancy. Qualitative analysis was done through coding of themes based on the Three Delays Framework. Descriptive analysis was used for quantitative data.

**Results:**

Male involvement in pregnancy and antenatal care in Magu district is low. Although men perceived antenatal care as important for pregnant women, most husbands had a passive attitude concerning their own involvement. Barriers for male involvement included: traditional gender roles, lack of knowledge, perceived low accessibility to join antenatal care visits and previous negative experiences in health facilities.

**Conclusion:**

Although several barriers impede male involvement during antenatal care, men’s internal motivation and attitudes towards their role during pregnancy was generally positive. Increasing community awareness and knowledge about the importance of male involvement and increasing accessibility of antenatal clinics can reduce some of the barriers.

**Electronic supplementary material:**

The online version of this article (doi:10.1186/s12884-016-0853-8) contains supplementary material, which is available to authorized users.

## Background

With an estimated 449 deaths per 100.000 live births, the maternal mortality ratio in Tanzania remains high [1]. Antenatal care (ANC), skilled birth attendance and access to emergency obstetric care is essential in the prevention of maternal deaths and severe acute maternal morbidity [2, 3]. Although most women in Tanzania attend at least one antenatal visit, few attend the recommended four visits and skilled birth attendance is estimated to remain below 50 % [4]. Although there is lack of evidence that male involvement will directly help reduce maternal deaths, their involvement has shown benefits for other maternal health outcomes and is therefore highly recommended by the World Health Organization (WHO) [5, 6]. Most research concerning male involvement has focused on their involvement during family planning, prevention of mother to child transmission of HIV and prevention of other sexually transmitted infections [7]. Although male involvement for these reasons is relevant during ANC, there are more reasons why male involvement is important. Since most men, in the majority of low-and middle-income countries, maintain a significant role in decision making in domains of private life, their role is highly influential in women’s choice for health care seeking behaviour. Therefore, men can encourage health facility visits, support good nutrition, reduce workload during pregnancy, assist in birth preparedness and provide emotional support [5, 8].

Several studies have attempted to increase male involvement during pregnancy, both at facility and community level. Facility level interventions include couples counselling and use of special education material targeting men [9–12]. Mass communication campaigns as well as house-visits targeting pregnant women and their partners are examples of interventions that aim to increase community knowledge and change traditional perceptions of male involvement [13–15]. Both type of interventions appear to positively contribute to increasing male involvement during pregnancy. However, few interventions appear to be sustainable, which is thought to be due to deep-rooted gender norms and health system factors which limit male involvement [8, 16].

The Tanzanian Ministry of Health, in line with WHO recommendations, also emphasises the particular importance of male involvement during ANC [4, 17]. Unfortunately, despite men’s positive attitudes, lack of male-friendly health infrastructure and limited understanding, by both community members and health facility staff about the role of men during pregnancy impedes their attendance [7, 10, 11, 18]. Maternal health interventions, aiming to increase health care seeking behaviour during ANC and birth, should pay attention to men’s social roles during pregnancy and local opportunities to join their partners for ANC visits.

This study was part of the Woman Centered Care Project (WCCP) of the African Woman Alliance. The WCCP aims to improve the quality of ANC through integrated interventions at facility and community level. Acknowledging the importance of men as partners and decision-makers, this study aimed to gain an understanding of male perceptions, attitudes and behaviour regarding their involvement in pregnancy and ANC, prior to implementation of the WCCP interventions in a rural district in Tanzania.

## Methods

### Study setting

The study took place in Magu district, Mwanza Region, between September 2013 and March 2014. Three quarters of the Tanzanian population lives in rural areas like Magu district, which has a population of approximately 300.000 inhabitants with 22,7 % of women of childbearing age. In rural Tanzania approximately 53 % of women deliver in health facilities and approximately 95 % of pregnant women attend ANC at least once, only 39 % having the recommended four visits [19]. These figures appeared to be similar in Magu district [20].

A mixed-method approach, including qualitative and quantitative methods, was used to enhance validity and reliability. Qualitative methods included observations, semi-structured interviews and focus group discussions (FGDs). Quantitative data was collected through a questionnaire distributed among women attending reproductive and child health care (RCH) clinics with the purpose of supplementing the qualitative data and giving additional information concerning male behaviour and attitudes.

### Qualitative data collection process

Observations were performed by EV and JB and included at least two full weekdays at six government health facilities, including the district hospital, two health centres and three dispensaries. Selection of facilities was based on different geographical locations in the district and previous findings regarding male attendance during exploratory research. Observations focussed on male attendance for ANC and human immunodeficiency virus (HIV) testing. Furthermore, in preparation of the interviews, informal conversations were held with facility staff regarding their services provision to couples during ANC clinics and other community members. Semi-structured interviews (*N* = 26) included three groups of men: those who accompanied their partners to ANC, those who did not accompany their partners to ANC and those whose partners did not attend ANC. Men were interviewed regarding personal experiences and perception of pregnancy and ANC, their perceived opportunities and barriers for involvement (Additional file 1). Most participants (men who accompanied their partners to ANC and men who did not accompany their partners to antenatal care) were purposefully selected at the ANC clinic. Remaining participants (husbands of women who did not attend ANC clinics) were identified through snowball sampling with the assistance of community (health) workers. Each FGD was attended by a minimum of 6 and a maximum of 12 participants and lasted between 100 to 180 min. The total of nine FGDs were of different composition, including two mixed gender FGDs, three ‘men only’ FGDs, two ‘women only’ FGDs as well as one FGD with traditional birth attendants (TBAs) (six women, one men) and one FGD with HCW involved in provision of ANC (five women, one men). During FGDs a variety of methods were used to facilitate discussion on topics which emerged during interviews, such as: vignettes, discussion of during interview frequently used quotes and photographs related to these topics. EV and JB conducted three FGDs together with the exception of six that were facilitated by EV and AK. Additional male and female note takers assisted to observe and report on non-verbal communication and group interaction processes.

### Quantitative data collection process

Women were approached to validate qualitative findings on male behaviour during pregnancy and to gain additional information. A questionnaire was developed based on the initial qualitative findings and a study by Byamugisha et al., who studied male involvement for prevention of mother to child transmission [21]. The questionnaire focussed on male involvement during pregnancy and included questions about them accompanying women to and attending at ANC, knowledge, physical support, spousal communication, financial support and their attendance for HIV testing. Questionnaires were translated in Kiswahili. Due to high illiteracy rates, fully self-administrated questionnaires were not possible; therefore AK assisted participants in the process. Quantitative data collection took place in four of the six different health facilities. The selection of these facilities was purposeful and determined by reported and observed level of male involvement; ‘high’ male involvement in Isolo dispensary versus ‘low’ involvement in Nyanguge Health Centre and two dispensaries in Lutale and Mwamabanza. Through convenience sampling the survey was conducted amongst 156 women waiting for RCH. Facilities were visited on a minimum of two different weekdays. This sample was calculated to be sufficient based on calculations made with OpenEpi (version 3) open source calculator. Refusal rate was not documented due to logistical challenges at the health facilities. Due to incompleteness 14 questionnaires were excluded from analysis.

### Data analysis

Qualitative data was transcribed verbatim in English by EV and subsequent analysis was performed with MAXQDA 11 (VERBI Software, Marburg, Germany). The ‘three delays model’ by Thaddeus and Maine (1994) was used to structure the analysis of the data. Factors that influence and delay male involvement exist at three levels: 1) delay in decision making influenced by men’s knowledge about pregnancy and their perceived role, socio-cultural barriers influenced by traditional gender norms, economic reasons such as costs for travel and income-generating activities and expectations and experiences about men’s ability to attend the clinic; 2) delay in reaching the facility influenced by transport issues, distance to the health facility and opening-hours of the health facility; 3) delay in receiving treatment due to unwelcoming facility infrastructure that is not designed to receive couples, unfriendly welcoming of staff, poorly staffed and equipped facilities [22]. A coding scheme was developed based on this model. Codes were then grouped according to categories and subcategories and discussed for analysis among the authors. Regrouping of categories was done to account for perspectives of different stakeholder groups. New themes emerged from the analysis and guided the interpretation of the data. Quantitative data was analysed with SPSS 20 (IMB Corp., Armonk, NY USA). Descriptive analysis was used and Chi-square tests were performed where possible, with associations considered significant at *P* <0,05. Low cell counts did not allow for effect modification and confounding.

### Ethical approval

Ethical approval for this research was granted by the National Institute of Medical Research (MR/53/100/103) in Tanzania and VU University Medical Centre (2013/135) in the Netherlands. Research permission was obtained from the Tanzanian Commission for Science and Technology. At community level the Magu district and village authorities gave permission for the data collection and introduced us in the communities and health facilities. All interview and FGD participants provided verbal consent and permission for audio recording.

## Results

### Qualitative results

A total of 26 men with an average age of 37 (range between 19 and 64) were interviewed: men who accompanied their partners (*N* = 9), men who did not accompany their partners (*N* = 9) and men of partners who did not attend antenatal care (*N* = 8). All men who accompanied their wives were from Isolo village and on average younger than other men. The nine FGDs had a total of 76 participants, including 40 men and 36 women. Participants had an average age of 33 and 26 respectively (range between 18 and 54). Despite their difference in involvement during ANC, the men did not differ much in attitudes and perceptions or behaviour concerning their involvement during pregnancy and ANC. Of the men whose wives did not attend ANC, responses varied, some did not know why she did not go, others tried to stimulate her to go and some did not see the benefit of going for ANC unless problems occurred.

### Male perception about pregnancy

Most men described pregnancy as something very common and not a particularly special moment in life. From their perspective, the ‘result’ of pregnancy was most important, with focus on delivery-related issues and less on the antenatal period. One man mentioned discussions with his wife were solely about issues surrounding delivery: *“Apart from delivery, nothing is being done and nothing is being discussed [with my wife] regarding pregnancy” (*50-year-old man during interview, Nyigogo)*.*

Although men described the pregnancy period to be a ‘normal’ event, some reported changes in the relationship with their wives. They felt women became ‘lazy’ and ‘demanding’, which started after women returned from ANC where they were given advice to restrain from heavy activities, to rest more often and to eat healthy food. Additionally, some reported pregnant women could be hostile towards their husband. Men admitted they had difficulty understanding this and acknowledged it could alter their own behaviour in a negative way. The following comment indicates some men seek the company of other women. *“When their wife is pregnant, many husbands spent time with other women, neglecting their own wife. That is how men behave”* (37-year-old woman during mixed-gender FGD, Lutale).

Most men acknowledged a healthy variety of food, reducing workload, and birth preparations are essential for pregnant women. Regarding responsibilities for providing these needs during pregnancy, men felt responsible, being traditional heads of the family, financial providers and decision makers. Although most men are aware that pregnant women should not perform heavy tasks, it seems apparent that cultural beliefs, gender roles and social stigma create barriers against this: *“I can’t do those household tasks, because I will be despised by the community and my relatives, so it is a shame for me”* (32-year-old man during FGD, Lutale).

Even though most men tended to have a passive role during pregnancy, stating pregnancy is ‘her burden’, according to HCWs, there is a growing awareness among men that pregnancy is a shared responsibility. One man illustrated this explaining: *“Pregnancy is a time to be very close to my wife, talking about plans and solving problems if they arise, calming her down”*. (47-year-old man during interview, Lutale).

### Male perception of ANC

Men were generally positive about their wives attending ANC. Even though some men do not know what is happening during ANC, most mention positive benefits such as disease prevention, testing of their wife’s and unborn child’s health and preparation for safe delivery. During FGDs men reported ‘not having to worry’ when their wife was attending ANC during pregnancy.

Even though TBAs are generally appreciated by men and often used in combination with formal ANC at health facilities, there is a general belief that facility services are better equipped and HCWs have more expertise. Some men considered it a routine responsibility to send their wives to ANC, while others considered it as specialised care, only needed in case problems arise. Some men reported they occasionally heard negative attitudes from HCWs towards women during ANC visits, reflecting maltreatment and disrespect, corruption, long waiting times, lack of available resources, and apathy about work.

Regarding men’s role joining women to ANC men responded that husbands traditionally are not involved in pregnancy-related issues, perceiving it as a ‘women only matter’: “*It is not part of our culture, it is western culture*” (29-year-old husband, during interview, Isolo). Additionally, they reported feeling uncomfortable when accompanying their wife to ANC and waiting for them outside: *“Nowadays women are struggling to involve us, by asking to accompany them to the clinic. But even if you arrive there, you don’t care and you may proceed with your own business (outside) instead of sitting with your wife and listening carefully to what it is being advised” (*54-year-old man during FGD, Lutale)*.* During FGDs it was often reported that lack of knowledge concerning pregnancy and related risk factors contributed to lack of awareness about the importance of their involvement with pregnancy-related issues. For example, men did not mention that an important reason for them to attend ANC was to be tested for HIV, neither did they mention the relevance of testing. Misconceptions about HIV testing during ANC, attributed to lack of information, contributed to men’s reluctance towards ANC attendance.

Respondents implicated in pregnancy care such as HCWs, TBAs and women participants confirm the importance of male involvement. They assert the importance of men participating in ANC citing the following reasons: ‘*they will hear the advice together, making them able to collaborate in pregnancy’*, ‘*husbands will be less ignorant of advice given by HCWs, knowing reduction of activities by their wives is not laziness*’, ‘*HIV testing involves both of them*’ (Responses during interviews and FGD’s)*.* Recent government policy developments are encouraging male attendance at ANC through the provision of preferential treament of women attending with their partner. In Magu district these policies have been taken up by HCWs. Despite this effort male attendance at ANC is extremely low at almost all health facilities. This low attendance can partly be explained by previously mentioned perceptions of men about their role during pregnancy, other contributory factors have also been described by both men and HCWs. First of all, not all HCWs appeared to inform women about the need for bringing their husband. Secondly, of those women who were informed, few shared this message with their husbands. Many men reported they were never invited to come to ANC with their wives. Some women assumed their husband’s lack of willingness to come or kept quiet to avoid arguments: “*Not even possible to tell him to come to ANC, because he will react badly”* (30-year-old woman during FGD, Mwamabanza). If a woman did inform her husband, they often made excuses or bluntly refused to join.

### Perceived accessibility of ANC

Accessibility of health facilities was influenced by lack of available transport, long distances and informal out-of-pocket expenses, causing barriers for both men and women to attend ANC. Secondly, most men stated they were unaware of their expected presence or they assumed that they were simply not welcome at ANC at all. Furthermore, one man reported that the fear of being tested for HIV at ANC is another barrier men experience: *“Fear for HIV makes husbands refuse to come to ANC, because they know they will get tested”* (48-year-old man during interview, Lutale).

Although current policy prioritises services at ANC for couples, some men felt that they were not welcomed and not received well: “*She [the HCW] was telling me* ‘*We only need women to test, what did you come to do here?’”* (38-year-old man during interview, Magu town). Another man stated: *“The nurses are just rude. They can ask you: ‘Are you also pregnant?’ So, go out!” (*51-year-old man during interview, Nyanguge). Additionally, waiting time and overall duration of ANC services were mentioned as negatively impacting on men’s ability to attend. Both during interviews and FGDs, farming commitments and other means of income-generation far away from home were also frequently mentioned as important reasons for low male attendance.

Men’s previous experiences at health facilities or the reputation of the health facility influenced their perception of the quality of care. Men reported several negative experiences such as feeling uncomfortable in the presence of HCWs who acted abusively towards their wives and distrust towards HCWs who ask for money: *“You can’t trust them, they are only after money. No professionalism!”* (28-year-old man during interview, Mwamabanza).

### Efforts to increase male involvement

Isolo dispensary, appeared to be the only health facility with relatively high male involvement. During observations in Isolo it appeared that a well-established, formal community rule exists which is strongly enforced by village and hamlet leaders. Men are encouraged to attend ANC with their wives and if they don’t, women will be denied service. One of the village leaders of Isolo said: *“Nowadays, if a woman is not accompanied by her husband [when she is visiting ANC], she is feeling ashamed”* (Village Executive Officer during informal conversation, Isolo). The rule appears to be widely known in this community and, compared to other health facilities, is effective in increasing the number of men attending ANC. During interviews and FGDs, however, perceptions, attitudes and behaviour of men in Isolo were not so different compared to men in other areas. Some men ignored the rule and refused to accompany their wife. Others, despite their attendance, did not fully understand the importance of their involvement: *“I am only going to attend with my wife because it is the rule, but I don’t know the purpose of it”* (36-year-old man during interview, Isolo). At the same time, a positive attitude towards male involvement during pregnancy is also visible in places were few men attend ANC and there is no formal rule in place: *“Personally, all the responsibilities of my wife are my responsibilities too, like cooking, washing the dishes or clothes. When my wife is pregnant or she has just delivered I help her in everything depending on her condition”* (30-year-old man, Kabila).

Most men were positive about increasing their knowledge and many were even explicitly asking for education concerning pregnancy-related issues. Education could make them *‘realise the importance of supporting their pregnant wi*ves’, ‘*become less ignorant’*, ‘*proactive instead of only reactive*’, ‘*finish with traditional gender roles’,* and *‘motivate them to attend ANC together’* (respondents during interviews and FGD’s)*.* Additionally, during certain FGDs and interviews, a number of men concluded that their own responsibilities such as work, were unacceptable excuses for their lack of involvement. Appropriate time management would increase chances of involvement. *“We have time, we only have to plan it”* (44-year-old man during FGD, Lutale).

### Quantitative results

Demographic characteristics of 142 women who completed the questionnaire are shown in Table 1 and results of the questionnaire are shown in Table 2. The majority of women (93,6 %) wanted their husbands to accompany them to ANC, both in Isolo and other health facilities. However, comparing Isolo and other health facilities, there is a significant difference of 88,4 % of women in Isolo reporting male accompaniment vs 20,4 % in other facilities (*P* <0,001). All the women from Isolo reported that their husband always paid informal payments for ANC or transport costs, compared to 84,6 % reported by women from other facilities. The percentage of husbands tested for HIV at ANC according to their wives was significantly different between Isolo and the other health facilities (*P* < 0,001).

**Table 1:** Demographic characteristics

*Characteristics*	*N* (%)
Age	
<20	14 (10)
20–40	119 (83)
>40	9 (7)
Tribe	
Sukuma tribe	132 (93)
Other tribe/not specified	10 (7)
Religion	
African Inland Church	38 (27)
Roman Catholic	42 (30)
Traditional religion/not specified	62 (43)
Marital status	
Single	8 (6)
Fiancée	2 (1)
Monogamous marriage	104 (73)
Polygamous marriage	23 (16)
Widow	2 (1)
Missing	3 (2)
Education of husband	
Primary school	97 (68)
Secondary school	17 (12)
No formal education	14 (10)
Missing	14 (10)
Household	
Living apart	6 (4)
Living together	125 (88)
Missing	11 (8)
Husband’s profession	
Farmer	105 (74)
Other	37 (26)

**Table 2:** Women’s perception on male involvement during pregnancy

	*Isolo facility* *N* (%)	*Other facilities* ^a^ *N* (%)
Number of questionnaires	44 (31)	98 (69)
*Husbands’ knowledge*		
*Knowledge about pregnancy* *(N = 133)*		
He has knowledge	22 (52,4)	36 (39,6)
He has knowledge, interested to know more	16 (38,1)	16 (17,6)
He has little to know knowledge	3 (7,1)	36 (39,6)
He has no knowledge, interested to know more	1 (2,4)	3 (3,3)
*Knowledge about wife’s ANC attendance* *(N = 133)*		
Yes, he sometimes knows	1 (2,4)	2 (2,2)
Yes, he always knows	38 (92,7)	88 (95,7)
No, he never knows	2 (4,9)	2 (2,2)
*Knowledge about content of ANC* *(N = 132)*		
Yes, husband has knowledge	41 (97,6)	71 (78,9)
Yes, husband has some knowledge	1 (2,4)	10 (11,1)
No, husband has no knowledge	0 (0,0)	9 (10,0)
*Husbands’ behaviour*		
*Involvement during pregnancy* *(N =131)*		
Yes, he is involved	36 (87,8)	66 (73,3)
He is a little involved	4 (9,8)	19 (21,1)
No involvement	1 (2,4)	5 (5,6)
*Payment for transport* *(N = 133)*		
Yes, he pays	42 (100)	77 (84,6)
No, he does not pay	0 (0,0)	14 (15,4)
Accompaniment to ANC* *(N = 142)*		
Yes, husband accompanies	39 (88,4)	20 (20,4)
No, nobody does or someone else accompanies	5 (11,6)	78 (79,6)
*Joins inside ANC room* *(N = 131)*		
Yes, he always joins inside	27 (64,3)	11 (12,4)
Yes, but only for HIV testing	11 (26,2)	9 (10,1)
Yes, sometimes	1 (2,4)	6 (6,7)
No, he was denied to enter	0 (0,0)	2 (2,2)
No, he never joins inside	3 (7,1)	61 (68,5)
HIV testing during ANC* *(N = 133)*		
Yes, he tested during ANC (at least once)	37 (81,1)	25 (27,5)
No, he never tested (during ANC)^b^	5 (11,9)	66 (72,5)
b. *HIV testing apart from ANC*		
Yes, he tested apart from ANC, at least once	3 (7,1)	26 (28,6)
No, he never tested	2 (4,8)	40 (43,9)
*Assistance at home for household tasks* *(N = 133)*		
By husband	22 (53,7)	23 (25,2)
By husband together with family (in law)	13 (31,7)	32 (35,2)
By family (in law)	5 (12,2)	17 (11,0)
By nobody	2 (4,7)	19 (20,9)
*Attendance during birth* *(N = 125)*		
Yes, he was present/available	38 (97,4)	66 (76,7)
No, he was not present	0 (0,0)	13 (15,1)
No, he was not present due to work	1 (2,6)	7 (8,1)
*Spousal discussions*		
*Discussions about ANC interventions or advice* *(N = 132)*		
Always	35 (85,4)	80 (87,9)
Sometimes	5 (12,2)	9 (9,9)
Never	1 (2,4)	2 (2,2)
*Decision making for place of delivery* *(N = 134)*		
Woman’s decision	2 (4,5)	27 (27,6)
Couple’s decision	30 (73,2)	46 (46,9)
Woman and family member’s decision	6 14,6)	13 (13,3)
Husband’s decision	3 (6,8)	7 (7,1)

Questionnaires results *(N = 142)*

*Significant *(p < 0,001)*

^a^Health Centre of Nyanguge and Dispensaries of Lutale and Mwamabanza *(N = 3)*

^b^HIV testing apart form ANC

## Discussion

The main finding of this study is the overall positive perception of men regarding the attendance of their partners at ANC, but passive attitude towards their own involvement, attributed mostly to external factors. This is similar to other studies showing a contradiction between men’s positive attitudes, while at the same time their participation and effort to be involved was low [23, 24]. In this study this contradiction was partly explained by men’s perspective of pregnancy as a socially constructed ‘female domain’, their lack of awareness of the importance of their involvement and perceived low accessibility to attend ANC clinics.

Examples of social stigma and traditional gender roles that appear to negatively influence male attendance in ANC have been reported in previous studies [8, 18, 21, 25]. Even though men might want to be more involved and some help their wives with household activities, they feel they can not publicly present themselves in such a way. Traditional gender roles and family structures remain important in Tanzania, influencing household practices and decision-making, also during pregnancy [18]. Efforts to increase male involvement can contradict with or challenge these roles and need to be taken into account, otherwise male involvement will remain focussed on financial support and decision-making [10, 11, 21, 24, 26].

Health system factors included poor attitude of HCWs towards men attending with their wives and this appeared to contribute to the perception some men had that they were not welcome at ANC. Long waiting time and long duration of ANC clinics were reported as well, however, this did not seem to be of major influence, similar to a study by Mullany [26]. Nevertheless, for male involvement to be possible, health facilities need to be more receptive and positive about the involvement of men. Appropriate policy, space and staffing inviting men to attend clinics and births would, according to some studies, increase the quality of care [7, 8, 27]. Possibilities for longer clinic hours, separate counselling rooms for couples and training of health care workers could be also considered, depending on the local needs [16]. Although HIV testing as part of ANC could be a barrier for male involvement, the quantitative analysis showed that with enforced male involvement more men would be tested as well. Appropriate information about the relevance of male involvement during ANC visits, beyond HIV testing as well as appropriate voluntary counselling and testing, could assist in reducing barriers.

With increasing government policies encouraging male involvement it is important to understand how this is translated into local practice. Enforcing rules to increase male involvement, such as occurs in Isolo village, seems effective in increasing male attendance and HIV testing indicating this could be a low-costs method to achieve better maternal and neonatal health outcomes. However, it is doubtful whether it leads to men understanding the importance of their presence. Although the majority of participants in Isolo village appeared to support local policy, perceptions and attitudes of men in Isolo were not obviously different from men in other locations. Unless services are directed towards men as well, it is unlikely that mere attendance will increase men’s understanding of their role during pregnancy and that interventions will be sustainable [16]. It also creates barriers for unmarried women to attend and as this is an already vulnerable group, such dictates may increase the risks they face and limit their access to services [8, 28]. Recommendations encouraging male attendance to ANC are important but must not undermine women’s choices and autonomy [5, 8, 27, 29]. The example of Isolo shows how national policies stimulating male involvement can be interpreted at local level with the danger of reinforcing gender inequalities and limiting women’s decision making power.

The example of Isolo teaches us that male involvement is not solely a matter of concern for HCWs but requires commitment and collaboration of the entire community in order to be successful [18, 19]. Increased awareness and understanding of both men and women regarding the importance of male-involvement and culturally acceptable interventions requires simultaneous community and facility based approaches [8]. Therefore, increasing HCW’s knowledge and providing them with additional tools, remains equally important, as HCWs have opportunities to motivate and educate couples attending ANC and make husbands feel more welcome through creating a more male-friendly environment [16, 30, 31].

Although this study is likely to represent situations in similar rural areas, limitations hinder our ability to generalize our findings. EV is a female foreign researcher and not fluent in the local language which possibly influenced understanding of local perceptions and practices. Active participation of other local researchers, however, helped the analysis of findings. During analysis of the questionnaires we did not correct for possible confounders. Health facilities are generally comparable as rural health facilities apart for the enforced male involvement in Isolo. Yet, it is possible that differences in staffing, availability of equipment or accessibility of the facilities could have influenced the results. However, qualitative data supports that the community rule was a strong driving force for male involvement and is likely to explain the results of the questionnaire. It is possible participants felt the researchers were representing government authorities and were perceived to support male involvement resulting in socially desirable answers, especially in Isolo village with its persuasive policies enhancing male involvement. Although the questionnaire was pre-tested, it is not a validated questionnaire. Additionally, it appeared difficult for women to answer the questions without help of a translator. Although we aimed for a representative sample of women to complete the questionnaire, results should be interpreted with caution as some women refused to participate. Additionally we excluded 14 questionnaires due to missing answers; possibly women were unable to complete the questionnaire due to clinic attendance, lack of time after the clinic visit or the decision to refuse to participate (at a later stage) because of feeling uncomfortable with the topic. Questionnaire findings were not fully consistent with qualitative findings as women reported more positively about men’s knowledge regarding pregnancy than men did themselves. However the mixed method design, concurrent triangulation and inclusion of different stakeholders have given valuable insights that can assist further research.

## Conclusion

Despite existing barriers and challenges to male involvement, men and women both seem supportive of increasing male involvement during pregnancy and attendance of men during ANC. With these positive perceptions, increased awareness of the importance of male involvement and the provision of tools to increase accessibility of men to clinics, there are ample opportunities to increase male attitudes and active participation during pregnancy. Although enforcing rules to increase male involvement provide additional challenges, they do seem to be effective in increasing both attendance and HIV testing of men during their partners ANC visit.

## Additional file

Additional file 1: Semi-structured Interview guide. (DOCX 31 kb)

## Abbreviations

ANC: antenatal care
HCW: health care worker
HIV: human immunodeficiency virus
FGD: focus group discussion
RCH: reproductive and child health are
TBA: traditional birth attendant
WCCP: Woman Centered Care Project
WHO: World Health Organisation
