# Supplementary material for: Opportunities for male involvement during pregnancy in Magu district, rural Tanzania
Source: BMC Pregnancy Childbirth. 2016 Mar 29;16:66. doi: 10.1186/s12884-016-0853-8 (PMC4810504; doi:10.1186/s12884-016-0853-8)
Supplement: Additional file 1: — Semi-structured Interview guide. (DOCX 31 kb) [file 12884_2016_853_MOESM1_ESM.docx]

- Semi-structured Interview guide -

Introduction including:

Expressing gratitude for participating, explanation about the aim of this interview and WCCP, confidentiality, anonymity, possibility to refuse and withdraw, permission to make an audio record and explicit informed consent. Emphasizing that there is not such a thing as a ‘wrong’ answer, mentioning when something is unclear and finally, asking if there are any questions before starting. Explaining role of interpreter.

Basic information of Participant

Name, date of birth, village

Introduction, pregnancy

1. Could you first of all tell me something about your situation at home?

- Family members, wife(s), children, parents

What do you do for a living?

Did you have some kind of education? If so, please specify.

1. How many times has your wife been pregnant?

Could you tell me about the first/last pregnancy of your wife?

Did the following/preceding pregnancies differ from the one you just explained to me? In what way?

- - How was it going?
  - What did she do/not do during pregnancy?
  - Complications? Birth preparation?
  - Where did she deliver?
  - How many of your children are still alive?

1. Could you describe to me what a women’s pregnancy means to you?
   - What implications has it for you, your marriage and family?
   - Is there something you fear about pregnancy? What, why? Etc.
2. What is important to be done and arranged during pregnancy? And why?
   - For example workload of pregnant woman, food, ANC visits, savings, birth preparations etc.
3. What people are involved with pregnancy? How and why?

Who are important during pregnancy? And why?

- - Who is supporting the your wife during pregnancy, who is giving her (informal) advise? Who does help her with heavy tasks?

1. What issues during pregnancy are/where you involved with?

- For example: decision making (about?), supporting in what way (financially/socially?), advise, avoiding her from doing heavy work, Place of birth etc.

What kind of pregnancy related issues do you talk about with you wife?

- - Decision making, tasks, support, food, suffering, birth preparations etc…?

*ANC*

1. Did your wife attended ANC? How many times when she is pregnant?

Why does she attend ANC?

What would you describe as good quality of ANC?

- - Services, attitude, equipment, knowledge etc.

1. Did you ever attended ANC together with your wife? Why? Did she ever ask you to attend?

How did the HCW react on your presence? What did you think?

- - How did you experienced (or what did you think of) that?)

1. Do you feel health workers try to involve husbands/men in ANC issues? What kind of?
   - Issues such as information about pregnancy, involvement in services, advice etc.

Do you consider male involvement important in ANC? How? Why (not)?

1. Have you ever visited a TBA with your wife? Why (not)?

How do TBA’s act towards you as a husbands/ towards husbands?

Is there any difference from the situation in the hospital?

1. Do you feel you have knowledge about pregnancy related issues (such as complications and danger signs)? Can you tell me some?

Where did you get this information?

1. If you would have any questions concerning ANC, where do you ask them?

Do you feel welcome to ask questions at the health facilities, to HCW or at TBA’s?

Do you ever feel shy or embarrassed by asking or speaking about pregnancy related issues?

- - Why? Because of what?

At what place, how and when would you like to get this information?

- - Could you think about something you think men/husbands would like to know more about?

1. Do you think it is important to be aware and to test and treat STI’s & HIV during pregnancy?
   - Why (not)?

Did you ever tested yourself for STI’s or HIV? Why (not)?)

1. If your pregnant wife is going for ANC and during checkup there is any kind of STI of HIV detected, what should to your opinion the Health services do?
   - Contamination, screening, treatment, awareness,

Do you think it would be a good thing to involve the husband? Why (not)?

Do you feel some kind of responsibility? How, why (not)?

*Delivery*

1. Where did your wife deliver?

Did you make any plans or preparations as to where to deliver beforehand? Which? Why (not)?

- - What preparations did you make? Were these preparations also used?

1. (Like you) some people have a delivery at home, together with a TBA. Why do people sometimes prefer that? Is delivery with a TBA helpful?
   - Did your wife ever delivered at home with a TBA? Could you tell me something about that? Who is present?)
2. Do you know people who delivered in a health facility?

Is delivery in a health facility helpful? If so, why don’t you/other people use the facilities (yourself)?

- - Who is present? Costs, distance, attitude of HCW, privacy or different from how you want it yourself?)

1. Where were you when your wife delivered? Why?

Did your wife ask you to come along? Why (not?)

- - Could you tell me more about this?

Over here it is not, but in some other countries it is normal for the husband to attend the delivery. What do you think about that?

- - Is that a good thing? Why (not)? Embarrassment? Support, empathy? What do you think your wife would prefer if it was possible in this community/culture?

*Role perception*

1. What is expected from a husband during the pregnancy of his wife in the community?

Do you ever criticize or feel criticized by people if you are helping your wife during pregnancy?

Do people ever tease you if you are helping your wife during pregnancy?

- - In what way? How do you respond? Does it bother you? Do you act differently because of it?

1. Are there pregnancy related issues husbands should not be involved with?
   - What kind of issues? Why not?
2. What do you feel women think about the role of men during pregnancy?

What do women expect form their husband during pregnancy?

- - What do they think about male involvement? Why?

1. Did you ever hear about women who did not tell their relatives or husband that they are pregnant? What do you know about it?
   - What could be a reason for not telling it? What do you think of this kind of situation if it happens? What kind of problems?

*Accessibility*

1. Do you, as husband, feel welcome in the health facilities (concerning ANC information about pregnancy or child health?

Have you ever seen posters that encourage couples to come together? Where, what did it say? Why?

- - Why do you think they encourage the pregnant woman to come together with her husband?

Should the health facilities be more accessible for men?

- - Why (not)? Do you have an example?

1. Does ANC during pregnancy also meet the needs of men?

Would you like to be involved in ANC issues? Why (not)? In what way?

How do you think it is possible to achieve more male involvement during pregnancy?

- - Information sessions (when, where?), possibility to ask questions, attendance during ANC services, village leaders, government etc.

We have reached the end of this interview. Is there anything you would like to add or comment or ask before we finish?

Thank you very much for you time! Nashukuru sana!
